# Glaucoma classification based on scanning laser ophthalmoscopic images using a deep learning ensemble method

**DOI:** 10.1371/journal.pone.0252339

**Published:** 2021-06-04

**Authors:** Dominika Sułot, David Alonso-Caneiro, Paweł Ksieniewicz, Patrycja Krzyzanowska-Berkowska, D. Robert Iskander

**Affiliations:** 1 Department of Biomedical Engineering, Wroclaw University of Science and Technology, Wroclaw, Poland; 2 Queensland University of Technology, Contact Lens and Visual Optics Laboratory, Centre for Vision and Eye Research, School of Optometry and Vision Science, Brisbane, Australia; 3 Department of Systems and Computer Networks, Wroclaw University of Science and Technology, Wroclaw, Poland; 4 Department of Ophthalmology, Wroclaw Medical University, Wroclaw, Poland; Massachusetts Eye & Ear Infirmary, Harvard Medical School, UNITED STATES

## Abstract

This study aimed to assess the utility of optic nerve head (onh) en-face images, captured with scanning laser ophthalmoscopy (slo) during standard optical coherence tomography (oct) imaging of the posterior segment, and demonstrate the potential of deep learning (dl) ensemble method that operates in a low data regime to differentiate glaucoma patients from healthy controls. The two groups of subjects were initially categorized based on a range of clinical tests including measurements of intraocular pressure, visual fields, oct derived retinal nerve fiber layer (rnfl) thickness and dilated stereoscopic examination of onh. 227 slo images of 227 subjects (105 glaucoma patients and 122 controls) were used. A new task-specific convolutional neural network architecture was developed for slo image-based classification. To benchmark the results of the proposed method, a range of classifiers were tested including five machine learning methods to classify glaucoma based on rnfl thickness—a well-known biomarker in glaucoma diagnostics, ensemble classifier based on inception v3 architecture, and classifiers based on features extracted from the image. The study shows that cross-validation dl ensemble based on slo images achieved a good discrimination performance with up to 0.962 of balanced accuracy, outperforming all of the other tested classifiers.

## Introduction

As the world’s population ages, glaucoma is becoming a leading cause of irreversible vision loss and blindness, with primary open-angle glaucoma (poag) being the most prevalent form of it [1]. Until it reaches an advanced stage, glaucoma is an asymptomatic disease, so methods of early diagnosis are of high importance [2]. Glaucoma diagnosis and management heavily rely on advanced imaging techniques, which typically image the *optic nerve head* (onh) and surrounding tissue [3]. The appearance of the onh is usually assessed with a fundus camera, but *scanning laser ophthalmoscopy* (slo) en-face imaging can also be utilized for differentiating glaucoma patients from normal subjects with high accuracy. For example, Haleem et al. [4] classified glaucoma patients based on geometric and non-geometric properties of different regions of the slo image, whereas Wollstein et al. [5] used the parameters of optics disk derived from slo images to differentiate early glaucoma patients from healthy individuals. Machine learning techniques have firmly entered the field of ophthalmology [6–9] and the number of studies showing their potential in glaucoma diagnosing is steadily growing [10–12]. These algorithms have also been used to differentiate early glaucoma patients from controls [13, 14]. However, most of those techniques focused on classifying glaucoma are based on information from visual field measurements, fundus camera images, or measurements of retinal nerve fiber layer (rnfl) thickness. The use of slo images, which are usually captured during the *optical coherence tomography* (oct) acquisition, for glaucoma classification using deep learning (dl) methods has not received, until recently, as much attention [15, 16].

dl methods are usually associated with large data volumes [17], where the overall performance of a classification system is highly dependent on the size of training data. However, for many applications within ophthalmology, data may be scarce. There exist methods to deal with the problem of insufficient sample size [18, 19], which include transfer learning [20], data augmentation [21], and model architecture modifications such as dropout [22]. Additionally, ensemble methods have been widely used to stabilize and improve the final model performance in the biomedical classification task [23]. The classifiers based on ensemble learning consist in the integration of multiple base classifiers, i.e., classifiers whose predictions had an impact on the final result. The goal is to create a model that will outperform all base classifiers included in its composition, whereas the effectiveness of such a model depends both on the diversity of its base classifiers [24] and on the proper choice of integration rule.

This study aimed to assess the utility of retinal slo images to support glaucoma diagnosis and to design a cross-validation ensemble of dl models that would accurately differentiate glaucoma patients from healthy controls in a low data regime.

## Methods

### Subjects and clinical measurements

The study was approved by the *Bioethical Committee of the Wroclaw Medical University* (kb–332/2015) and adhered to the tenets of the *Declaration of Helsinki*. Informed written consent to participate in the study was obtained from all subjects.

All subjects provided their medical history and underwent a comprehensive ophthalmic examination. In particular, *Goldmann applanation tonometry*, *slit lamp examination*, and *dilated stereoscopic examination* of the optic disc were performed for all subjects. *Visual field* (vf) parameters including *mean deviation* (md) and *pattern standard deviation* (psd) were measured using *standard automated perimetry* (*Humphrey Field Analyzer II 750; 24–2 Swedish interactive threshold algorithm; Carl Zeiss Meditec, Inc., Dublin*, ca). Additionally, spectral domain sd-oct (Spectralis, Heidelberg Engineering GmbH, Heidelberg, Germany) were acquired, using a circular scanning protocol around the optic nerve head to measure the average rnfl thickness as well as its mean value in six different onh sectors, that is temporal-superior (ts), temporal (t), temporal-inferior (ti), nasal-superior (ns), nasal (n) and nasal-inferior (ni). The oct instrument acquires an additional en-face slo image simultaneously during the acquisition of the oct-scan. Those images are used here for classification.

Subjects were excluded if they had a history of ocular surgery within 12 months before the onset of the study. Patients younger than 40 years old, with intraocular disease (e.g., macular degeneration, diabetic retinopathy, retinal vein occlusion) or neurological disorders affecting visual fields were also excluded from the study. Eyes with spherical equivalent of <−6.0 Diopters (d) or >+ 3.0 d, and cylinder correction of < − 3.0 d or >+ 3.0 d were also excluded. When both eyes met the inclusion criteria, the eye used for the study was randomly selected.

All the glaucoma patients in this study were clinically categorized as Poag type. Poag was defined as the persistence presence of glaucomatous optic nerve damage assessed using dilated stereoscopic examination of onh (i.e., concentric enlargement of the optic disc, rim thinning, or notching) with associated visual field defects in the presence of an open-angle. A normal visual field was defined as the absence of glaucomatous and neurologic field defects. Table 1 contains the group statistics of the clinical examination used to differentiate the two considered groups of subjects. The classification was performed by an experienced ophthalmologist (p.k.-b.).

**Table 1:** Mean values and standard deviations of the clinical parameters for the two considered groups of subjects together with the result of the Student’s t-test (p-values).

	control	glaucoma patients	*p*
n	122	105	—
age [years]	65 ± 9	68 ± 9	0.014
iop [mmhg]	17 ± 3	16 ± 3	0.083
vf md [db]	−0.38 ± 1.03	−9.78 ± 8.21	<0.001
vf psd [db]	1.63 ± 0.41	6.85 ± 4.09	<0.001
rnflav [*μ*m]	97 ± 8	62 ± 12	<0.001
rnfl ts [*μ*m]	136 ± 15	78 ± 22	<0.001
rnfl t [*μ*m]	70 ± 10	50 ± 14	<0.001
rnfl ti [*μ*m]	143 ± 18	73 ± 29	<0.001
rnfl ns [*μ*m]	106 ± 22	68 ± 18	<0.001
rnfl n [*μ*m]	73 ± 12	54 ± 15	<0.001
rnfl ni [*μ*m]	109 ± 20	70 ± 22	<0.001

n—size of the group; iop—intraocular pressure; vf—visual field; md—mean deviation; psd—pattern standard deviation; rnfl—retinal nerve fiber layer; av—average; ts—temporal-superior; t—temporal; ti—temporal-inferior; ns—nasal-superior; n—nasal; ni—nasal-inferior.

### Dataset

A total of 227 sd-oct slo images of 227 participants were used in this study. The participants were selected from consecutive patients who presented at the time of the study at the Outpatient and Glaucoma Clinic at the Department of Ophthalmology, Wroclaw Medical University. The dataset included 122 slo images of healthy control subjects and 105 images of glaucoma patients (see S1 Dataset). Additionally, the measurements of rnfl thickness were considered for comparison. The groups of glaucoma patients and healthy controls represent a valid sample from the general population. This has been ensured by examining the clinical parameters (see Table 1) that a trained ophthalmologist used for assigning a subject to a particular group. Therefore, it is assumed that the corresponding slo images from those subjects are also representative of the general population.

### Techniques

The dataset in this study contains en-face oct slo images. For the image classification task, a *Convolutional Neural Network* (cnn) was used, because cnn-based dl algorithms have proven in recent years to provide state-of-the-art performance for medical image classification tasks [25]. In ophthalmic applications, this model has already been applied to several image analysis applications, including retinal layer segmentation [26, 27], cone photoreceptor detection [28] and attention-based glaucoma detection [29]. dl algorithms generally require a relatively large amount of data to yield good classification results, which in the case of this study was infeasible. Thus, this limitation was considered during the initial stages of development.

**Fig 1:**
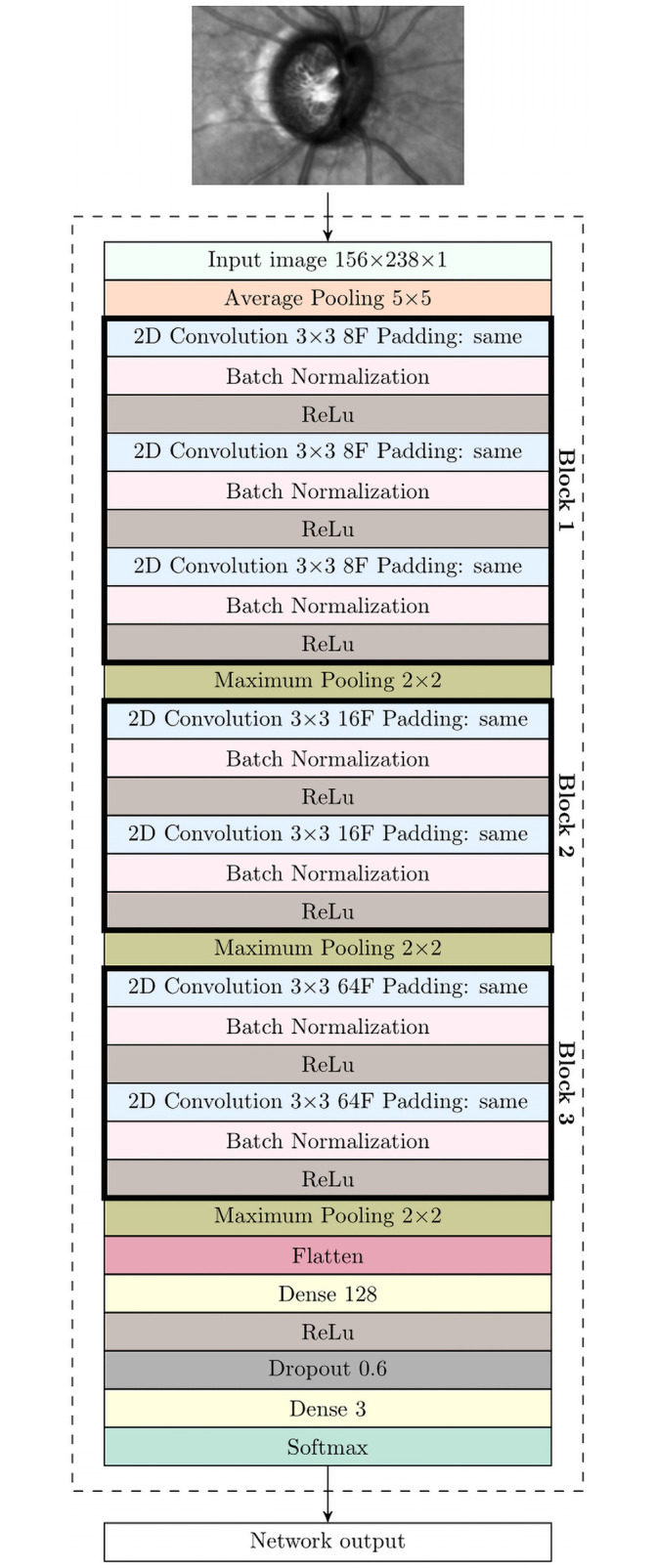
An overview of the classifier architecture. F indicates the number of filters and ReLu indicates the rectified linear unit.

Further, it was decided to design a task-specific cnn architecture with reduced complexity, with the aim of having fewer parameters required to train the model. The proposed neural network architecture is shown in Fig 1, with a cropped slo image of size 156×238 × 1 pixels being its input. Cropping corresponded to the instrument’s overlayed rectangular area and was performed to focus on the optic nerve head. The architecture consists of three major blocks, each of them containing 3, 2 and 2 convolution layers respectively, preceded by average pooling layer and followed by a maximum pooling layer. Following these blocks, there are two fully connected layers containing 128 and 2 units respectively. Between those layers, there is a dropout with a 0.60 rate, which aims to reduce the likelihood of overfitting. The last layer provides the result of the model, that is, the probability of an image belonging to a given class (healthy control and glaucoma subject). After every convolutional layer a batch normalization was performed using a *Rectified Linear Unit* (relu) activation function. For the first fully connected layer relu was utilized as activation function. To estimate the probability that a given image belongs to one of the two classes, the *softmax* function was used for the last and final layer, which provides the class with a higher probability selected as a model prediction.

For training purposes, the *Adaptive Moment Estimation* (Adam) optimizer [30] and *binary cross entropy* as the loss function were utilized. The models were trained up to 250 epochs. During training, the model with the best validation accuracy was saved and used later for testing. That occurred, in general, well before the 250th epoch. The learning rate was set to 0.001. The *Glorot uniform initializer* [31] was used for kernel and weights initialization, while the initial bias was set to zero. The software environment that was used for experimental evaluation consists of Keras 2.2.5 [32] with Tensorflow backend [33], Scikit-learn 0.20.3 [34] in Python 3.7.3.

To benchmark the proposed method performance with those of other networks, a modified inception v3 architecture [35] was also implemented. The original network, pre-trained on *Imagenet*, was used for the experiment, and the fully connected layers were removed, replacing them with two smaller fully connected layers of sizes 128 and 2, respectively. The training procedure, which includes the whole model, was identical to that of the custom architecture. The modification was introduced to improve the performance of the preliminary experiments on the original architecture.

Additionally to the dl methods a range of machine learning techniques were tested. This helps to assess the proposed whole-picture approach using dl, and its effectiveness in automatically extracting features for image classification versus the traditional machine learning that requires manual feature extraction methods. After extracting the features such as parameters obtained from *Principal Component Analysis* (pca) and gray-level co-occurrence matrix (glcm), a *Support Vector Machine* (svm) was used as a classifier.

Given that information on structural data (thickness) is commonly used to support glaucoma diagnosis, additional classifiers based on rnfl thickness values measured in six different sectors were trained. Hence, this analysis provides an extra layer of comparison for the proposed model. For this purpose, five supervised learning methods including *Multilayer Perceptron* (mlp), *k-Nearest Neighbors* classifier (knn), svm, *Classification and Regression Trees* (cart) and *Gaussian Naive Bayes* (gnb) were utilized.

The code used in the experiments is located on the Github platform and all the data used is available upon request (https://github.com/dsulot/slo_classification).

### Preprocessing of images

An original scan from oct Spectralis contains both the slo image of size 496 by 496 pixels, which provides the en-face 30°-view of onh, and the cross-sectional oct scans, which were not used for this study. A region of interest (roi) centered on the onh and of size of 156 × 238 × 1 pixels was used for analysis. An example of such slo image is shown in Fig 1. The image grey scale intensity within the roi was normalized between 0 and 1.

### Design of experiments

Because of limited data, it was decided to use data augmentation techniques for training purposes. Image transformations facilitate creating more training samples, prevent model overfitting and improve final accuracy. On observation of the image content, it was decided to use the following transformations: 1) horizontal/vertical flip, 2) shift (± 0.15 fraction of the total width/height), and 3) image rotation (± 50-degree range for random rotations). The parameters for each of these transformations were selected experimentally.

**Fig 2:**
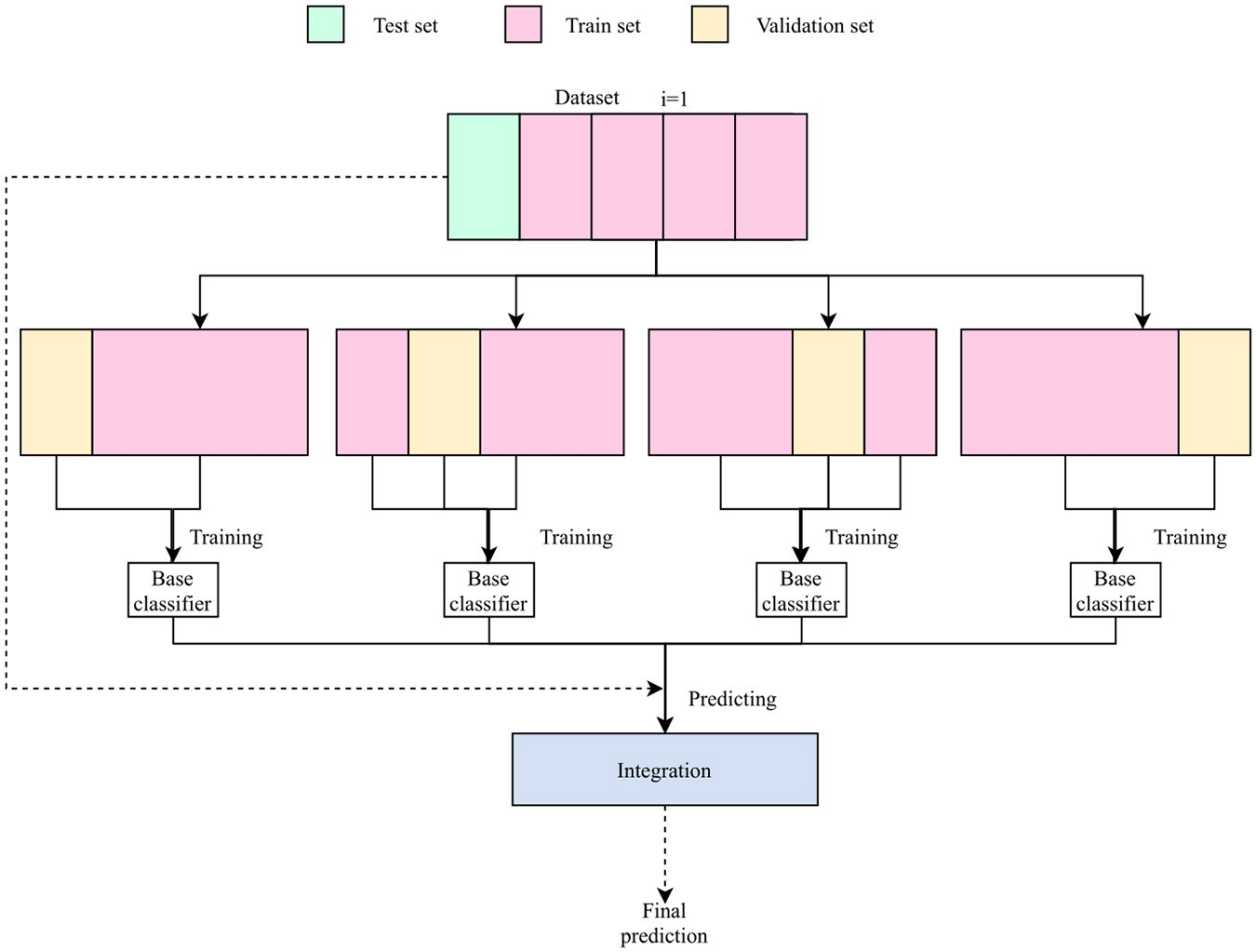
The k-fold cross-validation ensemble operation diagram for k = 5.

To check the stability of the proposed model and the model based on modified inception v3 architecture, *k-fold cross-validation* was utilized. The experiment was performed and conducted twice for different k values: 5 and 10. Within folds, to increase classification accuracy and stability, the ensemble of classifiers was created. The *cross-validation ensemble* was created from *k* − 1 classifiers trained on objects from train fold split into training and validation parts. Because each of the models was trained on a slightly different dataset (i.e., a different part of the training set was used to train and validate), they were able to establish different features to classify the images. An example scheme of a *5-fold cross-validation ensemble* is presented in Fig 2. The *cross-validation* protocol was also used for the other methods, including the rnfl thickness classifier and the classifier learned on the features extracted from images.

Finally, the effect of two types of classifier combination techniques with and without weighting was tested, including *majority voting* and *support accumulation* [36]. In a weighted approach, each classifier is weighted according to its performance that is calculated based on the validation dataset. The *balanced accuracy score* was used to evaluate the performance of the model and was defined as the mean between sensitivity and specificity [37]. Additionally, for all results, Wilcoxon test [38] was performed to assess whether the obtained results are statistically significantly different from each other.

## Experimental evaluation

### RNFL thickness-based classifiers

The results of glaucoma classification based on the rnfl thickness are considered because they represent one of the essential biomarkers in clinical diagnosis, whereas the information extracted from the slo images has a supplementary character, which is currently not utilized in the clinical practice. The scores obtained from the five considered standard classifiers based on rnfl thickness are presented in Table 2. It is evident that, in this case, mlp achieved the worst results, while the rest of the classifiers achieved similar balanced accuracy around 0.88. All of the considered methods are characterized by a relatively high standard deviation.

**Table 2:** Mean values and standard deviations of balanced accuracy across the five considered machine learning algorithms based on rnfl thickness. The number below the balanced accuracy metric, if any, indicates which model number obtained better and statistically significantly different results (Wilcoxon test, *α* = 0.05).

*k*	mlp	knn	svc	dtc	gnb
	1	2	3	4	5
5	0.721 ± 0.063	0.886 ± 0.065	0.880 ± 0.073	0.861 ± 0.058	0.886 ± 0.065
	—	1	1	1	1
10	0.749 ± 0.104	0.881 ± 0.086	0.894 ± 0.093	0.913 ± 0.075	0.888 ± 0.083
	—	1	1	1	1

mlp—multilayer perceptron, knn—k-nearest neighbors classifier, svm—support vector machine,

cart—Classification and regression trees, gnb—Gaussian naive Bayes

### Assessing the efficacy of image features

In recent years, dl methods have become the standard for image classification, yet machine learning methods (features extracted from image + classifiers) have shown to also provide a good image classification performance. Therefore, it was decided to check the performance achieved by the traditional machine learning algorithm. Table 3 shows the results of svm classifier trained using image features and trained on the whole image. Four techniques were considered: based on the vector created by averaging the image over columns and rows, the parameters calculated from glcm, the results from pca, and the combination of glcm and pca. After preliminary experiments, contrast and dissimilarity were used for further analysis from the available parameters calculated based on glcm. For the pca method, 99% of the explained variance was used (the image was initially flattened into a vector). The results indicate that this approach could obtain a balance accuracy metric up to 0.786 with the use of combined parameters from glcm and pca, which overall is inferior to the metrics from the rnfl thickness classifier. Within the considered techniques, there are no statistically significant differences in the results.

**Table 3:** Mean values and standard deviations of balanced accuracy across the five considered techniques: Classifier based on whole image as a vector, classifier based on glcm parameters, classifier based on averaged image over columns and rows, classifier based on pca results from an image, and classifier based on the combination of the pca results and the glcm parameters. The number below the balanced accuracy metric, if any, indicates which model number obtained better and statistically significantly different results (Wilcoxon test, *α* = 0.05).

*k*	whole image	averaged image	glcm	pca	glcm with pca
	1	2	3	4	5
5	0.770 ± 0.047	0.734 ± 0.088	0.694 ± 0.108	0.768 ± 0.047	0.786 ± 0.043
	—	—	—	—	—
10	0.777 ± 0.083	0.755 ± 0.098	0.695 ± 0.119	0.760 ± 0.067	0.770 ± 0.074
	—	—	—	—	—

### Assessing the potential of well-known, pre-trained architecture

In this experiment, it was decided to check not only the effect of well-known, pre-trained cnn architecture but also further its connection to ensemble learning. The results from a 5 and 10-fold cross-validation ensemble using the modified inception v3 architecture, as well as the results from the single inception v3 model, are presented in Table 4. The preliminary experiments have shown that a fine-tuned model on slo images for 10 epochs provided poor performance (0.492 ± 0.017 for a single model). Because of that, all models were fine-tuned for 250 epochs, like the models with custom architectures. As anticipated, the experiments indicate that ensemble learning improves the classification quality for each type of combination technique, providing statistically significant better metrics in the results. The obtained results show that for the modified, well-known architecture with transfer and ensemble learning, the model can classify glaucoma with a balanced accuracy of 0.945 based on slo images only.

**Table 4:** Mean values and standard deviations of balanced accuracy across different dl approaches based on slo images using modified inception v3 architecture. The number below the balanced accuracy metric, if any, indicates which model number obtained better and statistically significantly different results (Wilcoxon test, *α* = 0.05).

	single cnn model	ensemble methods			
*k*		majority voting		support accumulation	
		regular	weighted	regular	weighted
	1	2	3	4	5
5	0.909 ± 0.044	0.945 ± 0.042	0.926 ± 0.055	0.930 ± 0.050	0.930 ± 0.050
	—	—	—	—	—
10	0.877 ± 0.058	0.920 ± 0.050	0.920 ± 0.050	0.920 ± 0.050	0.920 ± 0.050
	—	1	1	1	1

### SLO-based classifier

Table 5 presents the overall classification performance of the considered dl methods for slo images using the custom cnn architecture. The mean value and the standard deviation of balanced accuracy are given for the individual single cnn models (to contrast those against the proposed ensemble model) as well as for classifier ensemble with different combination techniques. In each case, the classifier ensemble achieved better results than those of the single model. The ensemble classifier combined by majority voting achieved statistically significantly better results than those of the individual model and ensemble classifiers based on support accumulation. Overall, the results of classifier ensemble using a 5-fold cross-validation reached only a marginally superior balanced accuracy than that using a 10-fold one. However, the 5-fold cross-validation showed a better (smaller) standard deviation.

**Table 5:** Mean values and standard deviations of balanced accuracy across different dl approaches based on slo images using task-specific architecture. The number below the balanced accuracy metric, if any, indicates which model number obtained better and statistically significantly different results (Wilcoxon test, *α* = 0.05).

	single cnn model	ensemble methods			
*k*		majority voting		support accumulation	
		regular	weighted	regular	weighted
	1	2	3	4	5
5	0.905 ± 0.023	0.962 ± 0.016	0.930 ± 0.028	0.931 ± 0.015	0.931 ± 0.015
	—	1, 4, 5	—	—	—
10	0.893 ± 0.076	0.930 ± 0.070	0.930 ± 0.070	0.930 ± 0.070	0.930 ± 0.070
	—	—	—	—	—

Assessing the accuracy metrics, it is evident that information contained in the relatively low-resolution slo images can be successfully used for supporting glaucoma diagnosis. The dl methods reached accuracy of 0.962. The weighted ensemble models achieved almost identical results to those by regular models indicating that using this approach has no particular advantage, at least for the considered set of slo images. Additionally, Table 6 shows the mean values of the performance characteristics calculated across the folds obtained from the confusion matrix for all presented models together with the resulting sensitivity and specificity. It is evident that all ensemble models achieve high performance levels.

**Table 6:** Mean values of the performance characteristics across different dl approaches based on slo images using task-specific architecture.

k	method		tn	fp	fn	tp	sen	spe
5	single cnn model		21.6	2.8	1.6	19.4	0.924	0.885
	mv	regular	22.7	1.7	0.0	21.0	1.000	0.930
		weighted	22.6	1.8	1.4	19.6	0.933	0.926
	sa	regular	22.4	2.0	1.2	19.8	0.943	0.918
		weighted	22.4	2.0	1.2	19.8	0.943	0.918
10	single cnn model		11.0	1.2	1.2	9.3	0.886	0.902
	mv	regular	11.2	1.0	0.6	9.9	0.943	0.918
		weighted	11.2	1.0	0.6	9.9	0.943	0.918
	sa	regular	11.2	1.0	0.6	9.9	0.943	0.918
		weighted	11.2	1.0	0.6	9.9	0.943	0.918

tn—true negative, fp—false positive, fn—false negative, tp—true positive, sen—sensitivity, spe—specificity,

mv—majority voting, sa—support accumulation.

While comparing the results using custom architecture and the pre-trained, modified inception v3 architecture, it can be seen that for relatively small data sets, creating a compact, tailored architecture can be sufficient to achieve high classification accuracy and to reduce the time of experiments by using a smaller model that is faster to train. In each case, the classifier ensemble achieved better results with the task-specific architecture than with the modified, pre-trained well-known inception v3.

## Conclusion

In this study, the development of an ensemble of cnn models to classify en-face non-structural slo images into two different categories (glaucoma patients and healthy control subjects) were proposed. Despite the relatively low data regime and, consequently, the relatively small dataset to train the model, the results demonstrate that separation of the two considered groups could be performed with high accuracy using cross-validation ensemble of dl models (balanced accuracy up to 0.962).

Given the results presented in this paper, it is evident that the slo image contains valuable clinical information. Thus, this imaging modality combined with dl methods can support glaucoma diagnosis. Additionally, it is worth noting that for our dataset the classifiers based on rnfl thickness show an inferior performance. The thickness data is extracted from a single circular B-scan around the onh that may not be detailed enough to capture the structural changes in this cohort of glaucoma subjects. While comparing the traditional machine learning methods with the dl techniques, it is evident that ml method, as expected, showed an inferior performance to that of the proposed dl method. Regrading the dl solution, the findings demonstrate it was beneficial to develop a customized network architecture for this problem. The combination of slo (non-structural) and oct derived thickness data (structural) in a multi-modal dl approach should be considered in the future to further improve classification accuracy.

Given the limited dataset size, it is expected that by increasing it in future studies, the classification performance may be further improved. One of the limitations of working in a small data regime is a potential overfitting. Every effort has been made to prevent this, among other things, by using dropout layers and augmentation as well as by applying cross-validation which primarily shows that the results are repeatable at a similar level regardless of the test and training parts. The results for 5 and 10-fold cross-validation do not substantially vary. Additionally, generative adversarial methods to generate synthetic slo images can be used for more complex data augmentation purposes and improvement of model performance [39]. They will be explored in the future. It is worth noting that the slo images used in this study are captured as part of a standard oct scan. These en-face images are commonly used to check for measurement alignment within the retina or to track thickness changes in the follow-up studies. Although the slo image is embedded in oct, it is not normally used by clinicians for patient screening. However, several studies have shown the potential of slo imaging for glaucoma diagnosis, particularly for differentiating glaucoma patients from normal subjects [4, 5]. This study supports such developments with matching or increasing classification accuracy. Adding that the use of a smaller, task-specific architecture can be beneficial for classifying small data sets.

Finally, this study shows that dl methods based on an ensemble of classifiers can provide balanced accuracy to discriminate onh slo images of healthy and glaucoma patients, even in the low data regime. Given that this imaging modality is normally captured along with oct images, it can be relatively easily utilized for supporting glaucoma detection. Hence, onh slo images have the clinical utility to support glaucoma detection and management.

## Supporting information

S1 Dataset The data used in this study. (ZIP)

## References

[1] Quigley HA, Broman AT. The number of people with glaucoma worldwide in 2010 and 2020. British J. Ophthalmol. 2006;90(3):262–267. doi: 10.1136/bjo.2005.081224 PMC1856963 16488940

[2] Tatham AJ, Medeiros FA, Zangwill LM, Weinreb RN. Strategies to improve early diagnosis in glaucoma. Prog. Brain Res. 2015;221:103–133. doi: 10.1016/bs.pbr.2015.03.001 26518075

[3] Greenfield DS, Weinreb RN. Role of optic nerve imaging in glaucoma clinical practice and clinical trials. Am. J. Ophthalmol. 2008;145(4):598–603. doi: 10.1016/j.ajo.2007.12.018 18295183 PMC2367109

[4] Haleem MS, Han L, Van Hemert J, Fleming A, Pasquale LR, Silva PS, et al. Regional image features model for automatic classification between normal and glaucoma in fundus and scanning laser ophthalmoscopy (slo) images. J. Med. Syst. 2016;40(6):132. doi: 10.1007/s10916-016-0482-9 27086033 PMC4834108

[5] Wollstein G, Garwey-Health DF, Hitchings RA. Identification of early glaucoma cases with the scanning laser ophthalmoscope. Ophthalmology. 1998;105(8):1557–1563. doi: 10.1016/S0161-6420(98)98047-2 9709774

[6] Rahimy E. Deep learning applications in ophthalmology. Curr. Opin. Ophthalmol. 2018; 29(3):254–260. doi: 10.1097/ICU.0000000000000470 29528860

[7] Hogarty DT, Mackey DA, Hewitt AW. Current state and future prospects of artificial intelligence in ophthalmology: a review. Clin. Experiment. Ophthalmol. 2019;47(1):128–139. doi: 10.1111/ceo.13381 30155978

[8] Li X, Shen L, Shen M, Tan F, Qiu CS. Deep learning based early stage diabetic retinopathy detection using optical coherence tomography. Neurocomputing. 2019;369:134–144. doi: 10.1016/j.neucom.2019.08.079

[9] He X, Fang L, Rabbani H, Chen X, Liu Z. Retinal optical coherence tomography image classification with label smoothing generative adversarial network. Neurocomputing. 2020;405:37–47. doi: 10.1016/j.neucom.2020.04.044

[10] Chan K, Lee TW, Sample PA, Goldbaum MH, Weinreb RN, Sejnowski TJ. Comparison of machine learning and traditional classifiers in glaucoma diagnosis. IEEE. Trans. Biomed. Eng. 2002;49(9):963–974. doi: 10.1109/TBME.2002.802012 12214886

[11] Bowd C, Goldbaum MH. Machine learning classifiers in glaucoma. Optom. Vis. Sci. 2008; 85(6):396–405. doi: 10.1097/OPX.0b013e3181783ab6 18521021

[12] Kim SJ, Cho KJ, Oh S. Development of machine learning models for diagnosis of glaucoma. PLoS One. 2017;12(5):e0177726. doi: 10.1371/journal.pone.0177726 28542342 PMC5441603

[13] Sugimoto K, Murata H, Hirasawa H, Aihara M, Mayama C, Asaoka R. Cross-sectional study: Does combining optical coherence tomography measurements using the ‘Random Forest’ decision tree classifier improve the prediction of the presence of perimetric deterioration in glaucoma suspects?. BMJ Open. 2013;3(10):e003114. doi: 10.1136/bmjopen-2013-003114 PMC3796272 24103806

[14] Asaoka R, Murata H, Hirasawa K, Fujino Y, Matsuura M, Miki A, et al. Using deep learning and transfer learning to accurately diagnose early-onset glaucoma from macular optical coherence tomography images. Am. J. Ophthalmol. 2019;198:136–145. doi: 10.1016/j.ajo.2018.10.007 30316669

[15] Christopher M, Bowd C, Belghith A, Goldbaum MH, Weinreb RN, Fazio MA, et al. Deep learning approaches predict glaucomatous visual field damage from OCT optic nerve head en face images and retinal nerve fiber layer thickness maps. Ophthalmology. 2020;127(3):346–356. doi: 10.1016/j.ophtha.2019.09.036 31718841 PMC8063221

[16] Masumoto H, Tabuchi H, Nakakura S, Ishitobi N, Miki M, Enno H. Deep learning classifier with an ultrawide-field scanning laser ophthalmoscope detects glaucoma visual field severity. J. Glaucoma. 2018;27(7):647–652. doi: 10.1097/IJG.0000000000000988 29781835

[17] Al-Jarrah OY, Yoo PD, Muhaidat S, Karagiannidis GK, Taha K. Efficient machine learning for big data: A review. Big Data Res. 2015;2(3):87–93. doi: 10.1016/j.bdr.2015.04.001

[18] Liu TA, Ting DS, Paul HY, Wei J, Zhu H, Subramanian PS, et al. Deep learning and transfer learning for optic disc laterality detection: Implications for machine learning in neuro-ophthalmology. J. Neuroophthalmol. 2019; 40(2):178–184. doi: 10.1097/WNO.0000000000000827 31453913

[19] Wang P, Shen J, Chang R, Moloney M, Torres M, Burkemper B, et al. Machine learning models for diagnosing glaucoma from retinal nerve fiber layer thickness maps. Ophthalmol. Glaucoma. 2019;2(6):422–428. doi: 10.1016/j.ogla.2019.08.004 32672575 PMC7368087

[20] Pan SJ, Yang Q. A survey on transfer learning. IEEE Trans Knowl. Data Eng. 2009; 22(10):1345–1359.

[21] Perez L, Wang J. The effectiveness of data augmentation in image classification using deep learning. arXiv preprint arXiv:1712.04621 [Preprint]. 2017 [cited 2021 May 19]. Available from: https://arxiv.org/abs/1712.04621

[22] Hinton GE, Srivastava N, Krizhevsky A, Sutskever I, Salakhutdinov RR. Improving neural networks by preventing co-adaptation of feature detectors. arXiv preprint arXiv:1207.0580 [Preprint]. 2012 [cited 2021 May 19]. Available from: https://arxiv.org/abs/1207.0580

[23] Qummar S, Khan FG, Shah S, Khan A, Shamshirband S, Rehman ZU, et al. A deep learning ensemble approach for diabetic retinopathy detection. IEEE Access. 2019;7:150530–150539. doi: 10.1109/ACCESS.2019.2947484

[24] Rokach L. Ensemble-based classifiers. Artif. Intell. Rev. 2010;33(1):1–39.

[25] Shen D, Wu G, Suk HI. Deep learning in medical image analysis. Annu. Rev. Biomed. Eng. 2017;19:221–248. doi: 10.1146/annurev-bioeng-071516-044442 28301734 PMC5479722

[26] Fang L, Cunefare D, Wang C, Guymer RH, Li S, Farsiu S. Automatic segmentation of nine retinal layer boundaries in OCT images of non-exudative AMD patients using deep learning and graph search. Biomed. Opt. Express. 2017;8(5):2732–2744. doi: 10.1364/BOE.8.002732 28663902 PMC5480509

[27] Hamwood J, Alonso-Caneiro D, Read SA, Vincent SJ, Collins MJ. Effect of patch size and network architecture on a convolutional neural network approach for automatic segmentation of OCT retinal layers. Biomed. Opt. Express. 2018;9(7):3049–3066. doi: 10.1364/BOE.9.003049 29984082 PMC6033561

[28] Cunefare D, Fang L, Cooper RF, Dubra A, Carroll J, Carroll S. Open source software for automatic detection of cone photoreceptors in adaptive optics ophthalmoscopy using convolutional neural networks. Sci. Rep. 2017;7(1):6620. doi: 10.1038/s41598-017-07103-0 28747737 PMC5529414

[29] Li L, Xu M, Liu H, Li Y, Wang X, Jiang L, et al. A large-scale database and a CNN model for attention-based glaucoma detection. IEEE Trans Med. Imaging. 2019;39(2):413–434. doi: 10.1109/TMI.2019.2927226 31283476

[30] Kingma DP, Ba J. Adam: A method for stochastic optimization. arXiv preprint arXiv:1412.6980 [Preprint]. 2014 [cited 2021 May 19]. Available from: https://arxiv.org/abs/1412.6980

[31] Glorot X, Bengio Y. Understanding the difficulty of training deep feedforward neural networks. Proc. AISTATS. 2010;9:249–256.

[32] Chollet F. Keras. 2015. Available from: https://keras.io

[33] Martín Abadi et al. TensorFlow: Large-scale machine learning on heterogeneous systems. 2015. Available from tensorflow.org.

[34] Pedregosa et al. Scikit-learn: Machine learning in Python, J. Mach. Learn. Res. 2011;12:2825–2830.

[35] Szegedy C, Vanhoucke V, Ioffe S, Shlens J, Wojna Z. Rethinking the inception architecture for computer vision. Proceedings of the IEEE Conference on Computer Vision and Pattern Recognition. 2016:2818–2826.

[36] Woźniak M, Graña M, Corchado E. A survey of multiple classifier systems as hybrid systems. Infor. Fusion. 2014;16.

[37] Brodersen KH, Ong CS, Stephan KE, Buhmann JM. The balanced accuracy and its posterior distribution, In 2010 20th International Conference on Pattern Recognition. IEEE. 2010:3121-3124.

[38] Alpaydin E. Introduction to machine learning. MIT Press. 2009:511.

[39] Kugelman J, Alonso-Caneiro D, Read S, Vincent S, Chen F, Collins M. Constructing synthetic chorio-retinal patches using generative adversarial networks. Digital Image Computing: Techniques and Applications. 2019:1–8.

